# Supplementary material for: Echinoderms provide missing link in the evolution of PrRP/sNPF-type neuropeptide signalling
Source: eLife. 2020 Jun 24;9:e57640. doi: 10.7554/eLife.57640 (PMC7314547; doi:10.7554/eLife.57640)
Supplement: Figure 1—source data 1. — Accession numbers of the precursor sequences used for the peptide alignments in Figure 1. [file elife-57640-fig1-data1.docx]

| **Precursor/peptide name** | **Species name** | **Accession number or reference** |
| --- | --- | --- |
| PrRP-like | *Asterias rubens* | QBB78493.1 |
| PrRP-like | *Acanthaster planci* | XP_022086679.1 |
| PrRP-like | *Amphiura filiformis* | (Zandawala et al. 2017) |
| PrRP-like | *Strongylocentrotus purpuratus* | XP_001176371.1 |
| PrRP-like | *Saccoglossus kowalevskii* | XP_002737055.1 |
| PrRP-like | *Saccoglossus kowalevskii* 2 | Personal communication |
| PrRP | *Homo sapiens* | AAH69284.1 |
| PrRP | *Python bivittatus* | XP_025027440.1 |
| PrRP | *Lepisosteus oculatus* | ALD51284.1 |
| PrRP | *Latimeria chalumnae* | XP_006008888.1 |
| PrRP | *Paramormyrops kingsleyae* | XP_023653061.1 |
| PrRP | *Petromyzon marinus* | ALD51287.1 |
| PrRP | *Branchiostoma floridae* | XP_002595875.1 |
| NPY/NPF | *Saccoglossus kowalevskii* | XP_002741972.1 |
| NPY/NPF | *Homo sapiens* | NP_000896.1 |
| NPY/NPF | *Gallus gallus* | [NP_990804.1](https://www.ncbi.nlm.nih.gov/protein/NP_990804.1?report=genbank&log$=protalign&blast_rank=1&RID=91GYMWZP014) |
| NPY/NPF | *Danio rerio* | NP_571149.1 |
| NPY/NPF | *Branchiostoma floridae* | XP_002609542.1 |
| NPY/NPF | *Monieza expansa* | MH347240.1 |
| NPY/NPF | *Schmidtea mediterranea* | ADC84429.1 |
| NPY/NPF | *Octopus bimaculoides* | XP_014777727.1 |
| NPY/NPF | *Crassostrea gigas* | XP_011448178.1 |
| NPY/NPF | *Lymnaea stagnalis* | CAB63265.1 |
| NPY/NPF | *Helobdella robusta* | XP_009026400.1 |
| NPY/NPF | *Platynereis dumerilii* | GBZT01002538.1 (predicted from mRNA) |
| NPY/NPF | *Priapulus caudatus* | XP_014681442.1 |
| NPY/NPF | *Anopheles gambiae* | XP_315165.3 |
| NPY/NPF | *Drosophila melanogaster* | XP_001953779 |
| NPY/NPF | *Stomoxys calcitrans* | XP_013099916.1 |
| NPY/NPF | *Zootermopsis nevadensis* | XP_021923461.1 |
| NPY/NPF | *Caenorhabditis elegans* | NP_495111.1 |

**Figure 1 – source data 1. Accession numbers of the precursor sequences used for the peptide alignments in Figure 1.**
