# Supplementary material for: Echinoderms provide missing link in the evolution of PrRP/sNPF-type neuropeptide signalling
Source: eLife. 2020 Jun 24;9:e57640. doi: 10.7554/eLife.57640 (PMC7314547; doi:10.7554/eLife.57640)
Supplement: Figure 2—source data 1. [file elife-57640-fig2-data1.docx]

**Figure 2 – source data 1.** Accession numbers of the sequences used for the gene structure analysis in Figure 2 and Figure 2 - figure supplement 1.

| **Gene name** | **Species name** | **mRNA** | **Protein** | **Genome** |
| --- | --- | --- | --- | --- |
| PrRP-like | *Acanthaster planci* | XM_022230987.1 | XP_022086679.1 | NW_019091356.1 |
| PrRP-like | *Strongylocentrotus purpuratus* | XM_001176371.3 | XP_001176371.1 | NW_011971016.1 |
| PrRP-like | *Saccoglossus kowalevskii* | XM_002737009.1 | XP_002737055.1 | NW_003134358.1 |
| PrRP-like | *Saccoglossus kowalevskii 2* |  | Personal communication | NW_003156735.1 |
| PrRP | *Homo sapiens* | BC069284.1 | AAH69284.1 | Whole genome accessible with NCBI SPLIGN tool |
| PrRP | *Gallus gallus* | NM_001082419.1 | NP_001075888.1 | Whole genome accessible with NCBI SPLIGN tool |
| PrRP | *Python bivittatus* | XM_025171672.1 | XP_025027440.1 | NW_006532108.1 |
| PrRP | *Danio rerio* | NM_001245985.1 | NP_001232914.1 | Whole genome accessible with NCBI SPLIGN tool |
| PrRP | *Branchiostoma floridae* | XM_002595829.1 | XP_002595875.1 | ABEP02025055.1 |
| NPY/NPF | *Saccoglossus kowalevskii* | XM_002741926.2 | XP_002741972.1 | NW_003156735.1 |
| NPY/NPF | *Homo sapiens* | NM_000905.4 | NP_000896.1 | Whole genome accessible with NCBI SPLIGN tool |
| NPY/NPF | *Gallus gallus* | NM_205473.1 | [NP_990804.1](https://www.ncbi.nlm.nih.gov/protein/NP_990804.1?report=genbank&log$=protalign&blast_rank=1&RID=91GYMWZP014) | Whole genome accessible with NCBI SPLIGN tool |
| NPY/NPF | *Danio rerio* | NM_131074.2 | NP_571149.1 | Whole genome accessible with NCBI SPLIGN tool |
| NPY/NPF | *Branchiostoma floridae* | XM_002609496.1 | XP_002609542.1 | NW_003101541.1 |
| NPY/NPF | *Octopus bimaculoides* | XM_014922241.1 | XP_014777727.1 | NW_014672493.1 |
| NPY/NPF | *Crassostrea gigas* | XM_011449876.2 | XP_011448178.1 | NW_011936732.1 |
| NPY/NPF | *Helobdella robusta* | XM_009028152.1 | XP_009026400.1 | NW_008705278.1 |
| NPY/NPF | *Priapulus caudatus* | XM_014825956.1 | XP_014681442.1 | NW_014577064.1 |
| NPY/NPF | *Anopheles gambiae* | XM_315165.4 | XP_315165.3 | NT_078266.2 |
| NPY/NPF | *Caenorhabditis elegans* | NM_062710.5 | NP_495111.1 | NC_003280.10 |
