# Supplementary material for: Echinoderms provide missing link in the evolution of PrRP/sNPF-type neuropeptide signalling
Source: eLife. 2020 Jun 24;9:e57640. doi: 10.7554/eLife.57640 (PMC7314547; doi:10.7554/eLife.57640)
Supplement: Figure 4—source data 1. [file elife-57640-fig4-data1.docx]

**Figure 4 – source data 1.** Accession numbers of the receptor sequences used for the phylogenetic analysis shown in Figure 4.

| **Species** | **Accession number** | **Receptor type based on findings of this study, with previously used names in brackets** |
| --- | --- | --- |
| *Xenopus tropicalis* | XP_004911210.1 | NPYR2 |
| *Latimeria chalumnae* | XP_014350978.1 | NPYR2 |
| *Homo sapiens* | NP_000901.1 | NPYR2 |
| *Gallus gallus* | NP_001026299.1 | NPYR2 |
| *Saccoglossus kowalevskii* | XP_006817255.1 | NPY/NPF-R |
| *Aplysia californica* | XP_005089627.1, XP_005089880.1 | NPY/NPF-R |
| *Lottia gigantea* | XP_009066442.1 | NPY/NPF-R |
| *Lymnaea stagnalis* | CAA57620.1 | NPY/NPF-R (GPR 105) |
| *Capitela teleta* | ELT88377.1, ELT98787.1 | NPY/NPF-R |
| *Platynereis dumerilii* | AKQ63068.1 | NPY/NPF-R (GPCR 62) |
| *Crassostrea gigas* | XP_011444490.1 | NPY/NPF-R |
| *Schmidtea mediterranea* | ANO39140.1 | NPY/NPF-R (NPY5) |
| *Schmidtea mediterranea* | ANO39130.1 | NPY/NPF-R (NPY1) |
| *Schmidtea mediterranea* | ANO39139.1 | NPY/NPF-R (NPY3) |
| *Schmidtea mediterranea* | ANO39141.1 | NPY/NPF-R (NPY6) |
| *Tribolium castaneum* | XP_008198436.1 | NPY/NPF-R |
| *Drosophila melanogaster* | NP_001246947.1 | NPY/NPF-R |
| *Bactrocera dorsalis* | XP_011202740.1 | NPY/NPF-R |
| *Aedes_aegypti* | XP_021693392.1 | NPY/NPF-R |
| *Caenorhabditis elegans* | NP_508234.2 | NPY/NPF-R (NPR-11) |
| *Pristionchus pacificus* | PDM84819.1 | NPY/NPF-R (NPR-11) |
| *Caenorhabditis elegans* | NP_001293732.1 | NPY/NPF-R (NPR-12) |
| *Pristionchus pacificus* | PDM75274.1 | NPY/NPF-R (NPR-12) |
| *Asterias rubens* | MH807444 | sNPF/PrRP-R |
| *Acanthaster plancii* | XP_022101544.1 | sNPF/PrRP-R |
| *Strongylocentrotus purpuratus* | XP_003725178.1 | sNPF/PrRP-R |
| *Apostichopus japonicus* | PIK36230.1 | sNPF/PrRP-R |
| *Crassostea gigas* | XP_011451552.1 | sNPF-R |
| *Platynereis dumerilii* | AKQ63001.1 | sNPF-R (NKY receptor) |
| *Capitela teleta* | ELT88594.1 | sNPF-R |
| *Schmidtea mediterranea* | ANO39142.1 | sNPF-R (NPY7) |
| *Schmidtea mediterranea* | ANO39143.1 | sNPF-R (NPY8) |
| *Schmidtea mediterranea* | ANO39131.1 | sNPF-R (NPY10) |
| *Schmidtea mediterranea* | ANO39144.1 | sNPF-R (NPY9) |
| *Drosophila melanogaster* | NP_524176.1 | sNPF-R |
| *Tribolium castaneum* | XP_966794.1 | sNPF-R |
| *Bombyx mori* | NP_001127707.1 | sNPF-R (GPR A10) |
| *Bombyx mori* | NP_001127742.1 | sNPF-R (GPR A7) |
| *Bombyx mori* | NP_001127708.1 | sNPF-R (GPR A11) |
| *Aedes aegypti* | AGX84998.1 | sNPF-R |
| *Priapulus caudatus* | XP_014669822.1 | sNPF-R |
| *Caenorhabditis elegans* | NP_508816.1 | sNPF-R (NPR1) |
| *Pristionchus pacificus* | PDM70653.1 | sNPF-R (NPR1) |
| *Caenorhabditis elegans* | NP_501701.2 | sNPF-R (NPR2) |
| *Caenorhabditis elegans* | CAB05681.1 | sNPF-R (NPR3) |
| *Caenorhabditis elegans* | NP_001300304.1 | sNPF-R (NPR4) |
| *Pristionchus pacificus* | PDM83853.1 | sNPF-R (NPR4) |
| *Caenorhabditis elegans* | CCD70460.1 | sNPF-R (NPR5) |
| *Pristionchus pacificus* | PDM73363.1 | sNPF-R (NPR5) |
| *Homo sapiens* | NP_004239.1 | PrRP-R |
| *Danio rerio* | NP_001034615.1 | PrRP-R |
| *Gallus gallus* | AAW30382.1 | PrRP-R |
| *Alligator mississipiensis* | XP_019341424.1 | PrRP-R |
| *Xenopus tropicalis* | XP_002940396.1 | PrRP-R |
| *Branchistoma belcheri* | XP_019646333.1 | PrRP-R |
| *Branchistoma floridae* | XP_002608333.1 | PrRP-R |
| *Saccoglossus kowalevskii* | XP_002740053.1, XP_006815575.1, XP_002738225.1 | PrRP-R |
| *Homo sapiens* | NP_057624.3 | GPCR83 |
| *Callorhinchus milii* | XP_007900281.1 | GPCR83 |
| *Gallus gallus* | AEO92092.1 | GPCR83 |
| *Takifugus rubripes* | XP_011617071.1 | GPCR83 |
| *Strongylocentrotus purpuratus* | XP_003729750.1 | GPCR83 |
| *Asterias rubens* | MG744509, MG744510 | Luqin-R |
| *Strongylocentrotus purpuratus* | XP_783326.1, XP_783390.1 | Luqin-R |
| *Saccoglossus kowalevskii* | XM_002731957.1, XM_002731958.1, XM_006813011.1, XM_002731956.1 | Luqin-R |
| *Aplysia californica* | XP_012937781.1 | Luqin-R |
| *Lottia gigantea* | XP_009064514.1, XP_009064591.1 | Luqin-R |
| *Lymnea stagnalis* | AAB92258.1 | Luqin-R |
| *Octopus bimaculoides* | XP_014786450.1 | Luqin-R |
| *Capitella teleta* | ELT96089.1 | Luqin-R |
| *Platynereis dumerilii* | KP420214.1 | Luqin-R |
| *Priapulus caudatus* | XP_014666446.1, XP_014678140.1 | Luqin-R |
| *Acyrthosiphon pisum* | XP_008178727.1, XP_003241610.1 | RYamide-R |
| *Tribolium castaneum* | HQ709383.1 | RYamide-R |
| *Aedes aegypti* | AGX85003.1 | RYamide-R |
| *Drosophila melanogaster* | P25931.2 | RYamide-R |
| *Caenorhabditis elegans* | NP_001023541.1 | Luqin-R |
| *Trichuris suis* | KFD65303.1 | Luqin-R |
| *Homo sapiens* | AAB20303.1, NP_001049.1, NP_001050.1 | Tachykinin-R |
| *Ciona intestinalis* | [XM_009863501.2](https://www.ncbi.nlm.nih.gov/nuccore/1087807896) | Tachykinin-R |
| *Asterias rubens* | MG744511, MG744512 | Tachykinin-R |
| *Strongylocentrotus purpuratus* | XP_011662258.1 | Tachykinin-R |
| *Octopus vulgaris* | BAD93354.1 | Tachykinin-R |
| *Aplysia californica* | XP_012936180.1 | Tachykinin-R |
| *Lottia gigantea* | XP_009062052.1 | Tachykinin-R |
| *Capitella teleta* | [ELT98449.1](https://www.ncbi.nlm.nih.gov/protein/443698473?report=genbank&log$=protalign&blast_rank=1&RID=KZMXS48U014) | Tachykinin-R |
| *Urechis unitinctus* | BAB87199.1 | Tachykinin-R |
| *Drosophila melanogaster* | FBtr0085507 | Tachykinin-R |
| *Tribolium castaneum* | XP_008194527.2 | Tachykinin-R |
