# Supplementary material for: Echinoderms provide missing link in the evolution of PrRP/sNPF-type neuropeptide signalling
Source: eLife. 2020 Jun 24;9:e57640. doi: 10.7554/eLife.57640 (PMC7314547; doi:10.7554/eLife.57640)
Supplement: Figure 4—figure supplement 1—source data 1. [file elife-57640-fig4-figsupp1-data1.docx]

| **Precursor/peptide name** | **Species name** | **Accession number or reference** |
| --- | --- | --- |
| PrRP-like | *Asterias rubens* | QBB78493.1 |
| PrRP-like | *Acanthaster planci* | XP_022086679.1 |
| PrRP-like | *Amphiura filiformis* | (Zandawala et al. 2017) |
| PrRP-like | *Strongylocentrotus purpuratus* | XP_001176371.1 |
| sNPF-type | *Crassostrea gigas* | EKC33711.1 |
| sNPF-type | *Lymnaea stagnalis* | AAV41057.1 |
| sNPF-type | *Platynereis dumerilii* | AEE25645.1 |
| sNPF-type | *Schmidtea mediterranea* | DAA33926.1 |
| sNPF-type | *Drosophila melanogaster* | NP_724239.1 |
| sNPF-type | *Bombyx mori* | NP_001127729.1 |
| sNPF-type | *Aedes aegypti* | XP_021700214 |
| sNPF-type | *Tribolium castaneum* | DAA34847.1 |
| sNPF-type | *Caenorhabditis elegans* flp3 | NP_509694.1 |
| sNPF-type | *Caenorhabditis elegans* flp15 | NP_499820.1 |
| sNPF-type | *Caenorhabditis elegans* flp18 | NP_508514.2 |
| sNPF-type | *Caenorhabditis elegans* flp21 | NP_505011.2 |

**Figure 4 – source data 2.** Accession numbers of the precursor sequences used for the peptide alignments in Figure 4 - figure supplement 1.
