# Supplementary material for: Echinoderms provide missing link in the evolution of PrRP/sNPF-type neuropeptide signalling
Source: eLife. 2020 Jun 24;9:e57640. doi: 10.7554/eLife.57640 (PMC7314547; doi:10.7554/eLife.57640)
Supplement: Figure 4—figure supplement 2—source data 1. [file elife-57640-fig4-figsupp2-data1.docx]

| **Gene name** | **Species name** | **mRNA** | **Protein** | **Genome** |
| --- | --- | --- | --- | --- |
| PrRP-like | *Acanthaster planci* | XM_022230987.1 | XP_022086679.1 | NW_019091356.1 |
| PrRP-like | *Strongylocentrotus purpuratus* | XM_001176371.3 | XP_001176371.1 | NW_011971016.1 |
| PrRP-like | *Saccoglossus kowalevskii* | XM_002737009.1 | XP_002737055.1 | NW_003134358.1 |
| PrRP-like | *Saccoglossus kowalevskii 2* |  | Personal communication | NW_003156735.1 |
| sNPF | *Crassostrea gigas* | FQ665026.1 | EKC33711.1 | JH819141.1 |
| sNPF | *Pomacea canaliculata* | XM_025228896.1 | XP_025084681.1 | NC_037591.1 |
| sNPF | *Tribolium castaneum* | XM_008200483.2 | XP_008198705.1 | Whole genome accessible with NCBI SPLIGN tool |
| sNPF | *Apis mellifera* | XM_003250107.4 | XP_003250155.1 | Whole genome accessible with NCBI SPLIGN tool |
| sNPF | *Drosophila melanogaster* | NM_165316.2 | NP_724239.1 | Whole genome accessible with NCBI SPLIGN tool |
| sNPF | *Caenorhabditis elegans* flp-3 | NM_077293.6 | NP_509694.1 | NC_003284.9 |
| sNPF | *Caenorhabditis elegans* flp-15 | NM_067419.3 | NP_499820.1 | NC_003281.10 |
| sNPF | *Caenorhabditis elegans* flp-18 | NM_076113.5 | NP_508514.2 | NC_003284.9 |
| sNPF | *Caenorhabditis elegans* flp-21 | NM_072610.6 | NP_505011.2 | NC_003283.11 |

**Figure 4 – source data 3.** Accession numbers of the precursor sequences used for the gene structure analysis in Figure 4 - figure supplement 2.
